# A Case for Trauma-Informed Dementia Care: The Emancipatory Power of Psychological Resilience and Trauma From COVID-19

**DOI:** 10.1093/geroni/igab046.350

**Published:** 2021-12-17

**Authors:** Adrienne Ione

**Affiliations:** Silver Linings Integrative Health, LLC, Tacoma, WA, Washington, United States

## Abstract

The dominant nonpharmacological therapeutic approach in dementia care is “person-centered.” The racial disparities in disease and diagnosis affliction, represents an urge to incorporate trauma sensitivity. Antipsychotic use rates among this population being substantially higher than two years ago, as well as increasing evidence of ACE scores being a significant risk factor in dementia development, we need to consider the emancipatory power of trauma-informed dementia care. Data were collected from 103 peer-reviewed journal articles. Person-centered care practices were analyzed for adherence to SAMHSA’s principles that guide a trauma-informed approach. Studies were rated on a Likert-scale in six areas: safety; trustworthiness and transparency; peer support; collaboration and mutuality; empowerment and choice; and cultural, historical & gender issues. It was observed that of the 103 search returns, 38 met study criteria. Within these data, it was observed that the majority of studies scored low on incorporation and adherence to SAMSHA’s trauma-informed approach. While a few studies mentioned the magnifying effects of a current traumatic event on preexisting vulnerabilities, no study highlighted the growth or flourishing potential in triggering past trauma and the current traumatic event offering an opportunity of mind-somatic integration. This paper proposes trauma-informed dementia practices consider: 1) intake and assessment to include a screening of ACE score; 2) evaluation of (re)integrating current losses into current developmental and lifespan phase; and 3) offering therapeutic support of current traumatic event (COVID-19) to metabolize past traumatic events in the context of current developmental and lifespan phase.

